# Sustainable Management of Potato Tuber Moths Using Eco-Friendly Dust Formulations During Storage in the Andean Highlands

**DOI:** 10.3390/insects17010086

**Published:** 2026-01-13

**Authors:** Alex Villanueva, Fernando Escobal, Héctor Cántaro-Segura, Luis Diaz-Morales, Daniel Matsusaka

**Affiliations:** 1 Estación Experimental Agraria Baños del Inca, Dirección de Supervisión y Monitoreo de las Estaciones Experimentales, Instituto Nacional de Innovación Agraria (INIA), Cajamarca 06004, Peru; 2 Dirección de Recursos Genéticos y Biotecnología, Centro Experimental La Molina, Instituto Nacional de Innovación Agraria (INIA), Lima 15024, Peru; 3 Dirección de Investigación y Desarrollo Tecnológico (DIDET), Instituto Nacional de Innovación Agraria (INIA), Lima 15200, Peru

**Keywords:** potato tuber moth, *Symmetrischema tangolias*, storage protection, *Bacillus thuringiensis*, agricultural lime, industrial talc, wood ash, Andean highlands

## Abstract

Postharvest losses caused by potato tuber moths severely impact storage in the Andean highlands, where reliance on synthetic insecticides poses sustainability and safety concerns. This study evaluated eco-friendly alternatives for protecting stored seed tubers of the widely adopted cultivar INIA 302 Amarilis in Cajamarca, Peru. In two storage facilities, a completely randomized block design compared four treatments: *Bacillus thuringiensis* plus talc (Bt-talc), talc, agricultural lime, and wood ash against an untreated control. Powders were applied at 50 g per 10 kg of tubers, and incidence, severity of damage, and live larvae were assessed over 150 days. Bt–talc consistently achieved the lowest damage. Incidence in Cochapampa was 16.8% ± 6.2 with Bt-talc, compared with 58.1% ± 3.9 in the control; in Sulluscocha, incidence was 25.5% ± 4.8 and 64.2% ± 3.0 for Bt-talc and the control, respectively. A similar pattern was observed for moth-damage severity in both localities. Live larvae per unit were also markedly lower with 1.3 ± 0.3 (Cochapampa) and 1.6 ± 0.6 (Sulluscocha) under Bt–talc. A single dusting with Bt–talc, or alternatively agricultural lime, offers effective, accessible, and sustainable control of potato tuber moths in high-Andean storage.

## Simple Summary

Potatoes are a key food in the Andean highlands of South America. Stored tubers often suffer heavy losses from small moth larvae that tunnel into the potatoes and make them unsuitable for eating or planting. Farmers commonly rely on chemical insecticides, which can be costly and raise health and environmental concerns. We tested four practical, low-cost options that can be applied directly to seed potatoes during storage: a powder made with the natural bacterium *Bacillus thuringiensis* mixed with talc (Bt-talc), talc, agricultural lime, and wood ash. Over five months in two high-altitude communities in Cajamarca, Peru, we compared these options with an untreated control. The Bt-talc powder consistently provided the best protection, keeping the proportion of damaged potatoes below 15% in Cochapampa and below 30% in Sulluscocha, and markedly reducing both the incidence and the severity of internal injury caused by moth larvae. Agricultural lime also performed well and is inexpensive and widely available in rural areas. These results show that simple, eco-friendly materials can help Andean families protect their potatoes in storage, reduce dependence on synthetic insecticides, and safeguard food and seed for the next planting season.

## 1. Introduction

Potato (*Solanum tuberosum* L.) is one of the most important staple crops worldwide, ranking third after rice and wheat in terms of human consumption [1]. Originating in the Andean region of South America, its domestication began approximately 8000 years ago, followed by a long process of diversification along the Andes, from northern Bolivia to western Venezuela [2]. Archaeological evidence indicates that the highland region between Peru and Bolivia represented a primary center of domestication around 5500 years ago [3]. Today, Peru is the leading potato producer in Latin America, with an annual output of 5.4 million tons and an average yield of 16.8 t ha^−1^ [4].

Within this national scenario, Cajamarca (6°24′ S; 78°48′ W) stands out as the fourth most important potato-producing region in Peru, with 28,627 ha cultivated, after Puno, Huánuco, and Cusco. The crop is distributed across twelve of its thirteen provinces, with the largest cultivated areas found in Cutervo, Chota, Cajamarca, and Celendín [5].

Cajamarca hosts a diverse portfolio of potato germplasm, including more than a dozen traditional cultivars of the Phureja group, as well as several improved varieties adopted throughout the region, such as Chachapoyana, Blanca, and Huagalina [6,7]. Among the varieties cultivated, INIA 302 Amarilis occupies a predominant position. Released in 1993 through collaborative work between the International Potato Center (CIP) and the National Institute of Agrarian Innovation (INIA) [8], Amarilis is recognized for its resistance to late blight (*Phytophthora infestans*), high yield potential, and desirable culinary quality, attributes that have facilitated its broad adoption in the northern highlands [9,10]. Nutritionally, each 100 g tuber contains approximately 78 g moisture, 18.5 g starch, and is rich in potassium (560 mg) and vitamin C (20 mg) [11]. The availability of certified seed and its consistent performance across diverse production zones have further reinforced its dominant role in Cajamarca’s potato systems.

Despite these agronomic and nutritional advantages, potato production and storage in the high Andes suffer persistent challenges from insect pests. Two species, the potato tuber moth *Phthorimaea operculella* (Zeller, 1873) and the Andean potato tuber moth *Symmetrischema tangolias* (Gyen, 1913), both belonging to the family Gelechiidae (Lepidoptera), are considered the most destructive during storage [12,13]. Their impact is particularly severe under traditional storage conditions without refrigeration, where infestation rates can range from 60% to 90% [14]. In the high Andes, these storage structures are non-climate-controlled facilities, and tubers are fully exposed to ambient fluctuations in temperature and humidity. Both pests exhibit nocturnal adult activity, high oviposition capacity (100–150 eggs per tuber), and larval penetration through buds, leading to gallery formation and deposition of frass inside the tuber [15]. These injuries not only compromise tuber quality but also increase susceptibility to fungal and bacterial infections, further reducing their marketability [16].

Traditionally, pest control during storage has relied heavily on chemical insecticides. Organophosphates such as chlorpyrifos and carbamates like carbaryl are among the most widely applied [17]. While effective in reducing postharvest losses [18], their prolonged use has raised serious concerns, including the development of resistant insect populations, environmental contamination, and health risks to farmers and consumers [19,20]. These limitations underscore the urgent need to explore safer and more sustainable pest management strategies.

In response, numerous studies have investigated ecological alternatives for controlling *S. tangolias* and *P. operculella* [21]. Promising results have been reported using *Bacillus thuringiensis* formulations and organic mulches such as dried *Lantana* leaves, both of which significantly reduced infestation rates [12]. Similarly, the application of 100 g of industrial Talc plus *Bacillus thuringiensis* (Bt-talc) per 25 kg of potato provided complete protection against tuber damage during a two-month storage period [22]. Other approaches combining *Bacillus thuringiensis* with botanical powders, such as *Artemisia* sp., also demonstrated substantial reductions in infestation levels compared to untreated controls [23].

Among inert dusts traditionally used by Andean farmers, agricultural lime and wood ash have attracted scientific attention due to their proven physical effects on potato tuber moths. Experimental evaluations by Kroschel and Koch [24] demonstrated that finely ground calcium carbonate (agricultural lime) causes high mortality of *Phthorimaea operculella* larvae through abrasion of the cuticle, removal of epicuticular waxes, and subsequent desiccation. Wood ash, although generally less potent than CaCO_3_, exhibits similar desiccating and abrasive properties and has historically been used as a natural protectant for stored potatoes in the Andes. These mechanisms support the inclusion of agricultural lime and wood ash as eco-friendly alternatives for postharvest moth management under highland storage conditions

In addition to biological controls such as *Bacillus thuringiensis*, several inert dusts have historically been employed as low-cost protectants for stored crops. Mineral powders—including lime (calcium hydroxide), limestone (calcium carbonate), dolomite, magnesite, and other rock-derived materials have been shown to reduce insect infestation by abrading the cuticle, absorbing cuticular lipids, and promoting larval desiccation (Golob, 1997) [25]. Early storage studies demonstrated that these minerals can provide substantial protection when applied at rates >10 g kg^−1^ of grain [26,27]. Wood ash, another widely available traditional material, has also exhibited notable insecticidal properties. Its efficacy depends on particle size and geochemical composition—typically enriched in CaO (37.6%) and properly prepared ash can achieve >90% mortality in storage pests such as *Sitophilus granarius* [28]. Moreover, additional evidence indicates that wood ash is effective across a broader range of systems; for example, Wahedi et al. [29] reported significant reductions in insect pest infestation in okra (*Abelmoschus esculentus*), spinach (*Spinacia oleracea*), and sorrel (*Rumex acetosa*) compared with untreated controls. Collectively, these findings provide a strong scientific foundation for evaluating agricultural lime and wood ash as accessible, eco-friendly postharvest protectants for smallholder potato storage systems in the Andes.

Considering these advances, the present study was designed to evaluate four eco-friendly alternatives for the control of *S. tangolias* under storage conditions in the Cajamarca region. By providing practical and evidence-based information, this work aims to contribute to the development of sustainable pest management strategies that can safeguard food security and support rural livelihoods in high-Andean communities.

## 2. Materials and Methods

### 2.1. Study Site

The study was conducted in two storage facilities located in the highlands of Cajamarca, Peru: Sulluscocha, Namora district (7°12′05.76″ S, 78°22′41.18″ W; altitude: 2950 m a.s.l.), and Cochapampa, Chetilla district (7°10′56.39″ S, 78°40′15.25″ W; altitude: 3100 m a.s.l.), both in the province and department of Cajamarca (Figure S1).

General climatic conditions for each site were obtained from nearby National Meteorology and Hydrology Service of Peru (SENAMHI) stations. A detailed description of monthly temperature, rainfall, and humidity trends during the 150-day storage period can be found in Figure S2.

### 2.2. Plant Material

According to Pradel et al. [30], the potato variety INIA 302 Amarilis is the most important in the Andean highlands of Peru. In Cajamarca, it is the most widely cultivated variety, covering 44% of the total planted area, while its national adoption rate is estimated at 7.15% [8,9]. The seed tubers used in this study were obtained from the Baños del Inca Agrarian Experimental Station (EEA, Baños del Inca, Peru), where they were selected, graded, and packed in polyethylene bags before being transported to the experimental sites.

### 2.3. Experimental Design

A total of 4050 seed tubers (300 kg) were stored across the two localities. The experiment was arranged in a completely randomized block design (CRBD), with five treatments—including four control alternatives and one control, with three replications per locality. Storage facilities were characterized by diffuse light, adequate ventilation, and dimensions of 2 m in width, 5 m in length, and 2.5 m in height. Each experimental unit consisted of wooden crates (0.25 × 0.30 × 0.70 m) containing 10 kg of seed tubers per crate, resulting in 15 experimental units per locality.

### 2.4. Experimental Management

For each experimental unit, 135 seed tubers were counted, equivalent to approximately 10 kg. Treatments were applied at a rate of 50 g per 10 kg of potato seed. Application consisted of placing the tubers into a thick plastic bag, adding the corresponding treatment, and carefully mixing until the product was evenly adhered to the tuber surface. The control units were handled in the same way; 135 seed tubers were counted and placed in plastic bags but no treatment material was added. Finally, the experimental units were placed inside the storage facilities under diffuse light conditions (Table 1).

### 2.5. Storage Variables Evaluated

The evaluation period lasted 150 days, from 29 August 2024, to 29 January 2025. Data were recorded at the end of the storage period and analyzed for each evaluated variable. Each treatment was assessed in three independent replicates (n = 3). Each replicate consisted of a crate containing 135 seed tubers (approximately 10 kg).

During the evaluations, only *Symmetrischema tangolias* was detected in the storage facilities. No larvae or adults of *Phthorimaea operculella* were recorded; therefore, all incidence, severity, and larval counts correspond exclusively to *S. tangolias*.

#### 2.5.1. Incidence of Moth Attack (%)

The number of tubers showing visible external damage caused by moth larvae—evidenced by holes in sprouts and surface galleries—was quantified. The incidence percentage was calculated according to Baca et al. [31] using the following Formula (1):

(1) $$Incidence\;(\%)=\frac{Number\;of\;damaged\;tubers}{Total\;number\;of\;tubers\;evaluated}\;\times 100$$

#### 2.5.2. Severity of Moth Damage (%)

To determine damage severity, affected tubers were cut into four equal parts, each representing 25% of the total tuber. The percentage of internal surface affected was visually estimated in each segment. The sum of the values obtained from the four segments was used to calculate the severity percentage (2), following Baca et al. [19]:

(2) $$Severity\;(\%)=\frac{\sum (\%\;of\;damaged\;area\;per\;tuber)}{Total\;number\;of\;tubers\;damaged}\;\times \;100$$

#### 2.5.3. Number of Live Larvae

The number of live moth larvae, considering all larval stages, present in the affected tubers was recorded.

### 2.6. Statistical Analysis

Statistical analyses were performed using InfoStat v.2020. Since the assumptions of normality and homoscedasticity were not satisfied, data were analyzed with the non-parametric Kruskal–Wallis test. When significant differences were detected, Dunn’s post hoc test with Šidák correction was applied (α = 0.05). All graphs and visualizations were produced using R software (version 4.5).

## 3. Results

### 3.1. Incidence of Moth Attack (%) in Sulluscocha and Cochapampa

**Figure 1:**
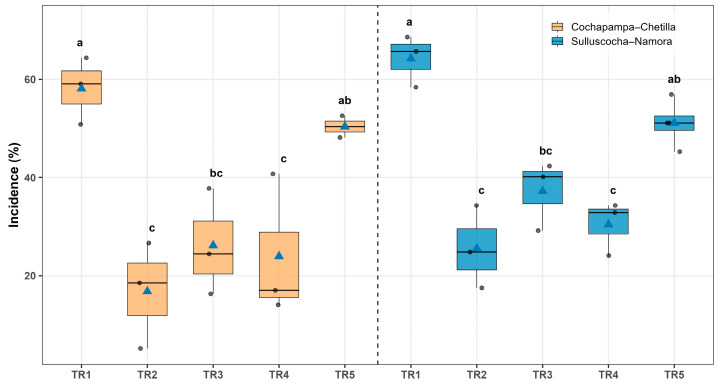
Incidence of potato tuber moth (*Symmetrischema tangolias*) damage in stored potato tubers under five treatments: TR1 (control), TR2 (*Bacillus thuringiensis* var. *kurstaki* plus industrial talc), TR3 (industrial talc), TR4 (agricultural lime), and TR5 (wood ash), evaluated in two localities: Cochapampa–Chetilla (orange) and Sulluscocha–Namora (blue). Boxes represent the interquartile range, the horizontal line inside each box indicates the median, blue triangles correspond to the mean, and grey dots represent individual observations. Different lowercase letters above the boxes denote significant differences among treatments according to the Šidák test (*p* < 0.05).

The incidence of potato tuber moth damage showed significant differences among treatments in both localities (Figure 1). In Cochapampa–Chetilla, the lowest incidence was recorded in treatment TR-2 (*Bacillus thuringiensis* var. kurstaki + industrial talc, 5 kg t^−1^ of seed), with 16.8% ± 6.2, followed by TR-4 with 23.95% ± 8.44%. In contrast, the control treatment (TR-1) exhibited the highest incidence (58.1% ± 3.9%), representing more than twice the level of damage observed in TR-2 (Figure 1, Table S1).

A similar trend was observed in Sulluscocha–Namora. Treatment TR-2 significantly reduced the incidence of damage (25.5% ± 4.8%), followed by TR-4 (30.4% ± 3.1%). Once again, the control treatment (TR-1) reached the highest incidence (64.2% ± 3.0%), nearly double the damage recorded in TR-2 (Figure 1, Table S1).

### 3.2. Severity of Month Damage (%) in Sulluscocha and Cochapampa

**Figure 2:**
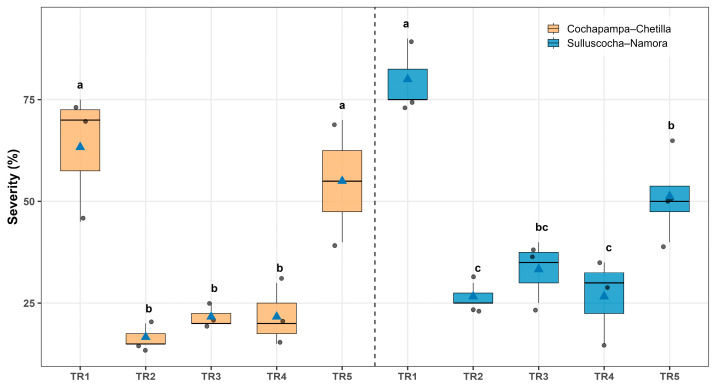
Severity of potato tuber moth (*Symmetrischema tangolias*) damage in stored potato tubers under five treatments: TR1 (control), TR2 (*Bacillus thuringiensis* var. *kurstaki* plus industrial talc), TR3 (industrial talc), TR4 (agricultural lime), and TR5 (wood ash), evaluated in two localities: Cochapampa–Chetilla (orange) and Sulluscocha–Namora (blue). Boxes represent the interquartile range, the horizontal line within each box indicates the median, blue triangles correspond to the mean, and grey dots show individual observations. Different lowercase letters above the boxes indicate significant differences among treatments according to the Šidák test (*p* < 0.05).

The severity of potato tuber moth damage differed significantly among treatments in both localities (Figure 2). In Cochapampa–Chetilla, treatment TR-2 (*Bacillus thuringiensis* var. *kurstaki* + industrial talc, 5 kg t^−1^ of seed) resulted in the lowest severity (16.6% ± 1.6%), followed by TR-3 (industrial talc) and TR-4 (agricultural lime), with 21.6% ± 1.6% and 21.6% ± 4.4%, respectively. In contrast, the control treatment (TR-1) recorded the highest severity (63.3% ± 9.2%), approximately three times greater than TR-2 (Figure 2, Table S1).

**Figure 3:**
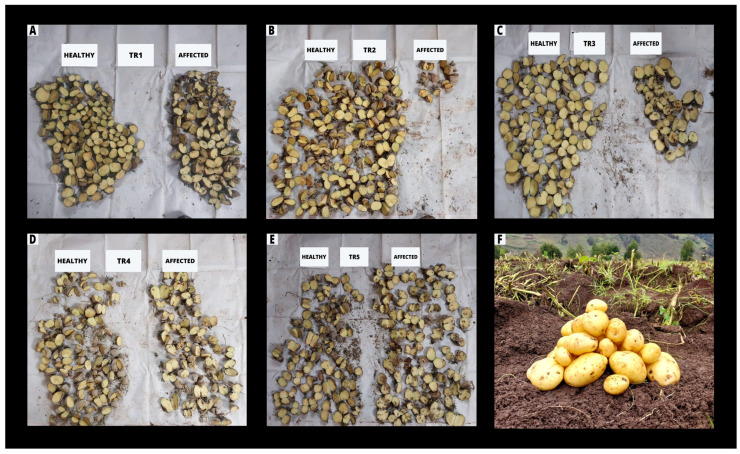
Condition of potato tubers (cv. INIA 302 Amarilis) after 150 days of storage under different treatments in Cochapampa, Cajamarca, Peru. Each panel shows healthy and affected tubers under five treatments: (**A**) TR1 (Control), (**B**) TR2 (*Bacillus thuringiensis* + talc), (**C**) TR3 (talc), (**D**) TR4 (agricultural lime), and (**E**) TR5 (wood ash). “Healthy” refers to undamaged tubers, while “Affected” indicates those showing internal or external damage caused by potato tuber moth larvae (*Symmetrischema tangolias*). (**F**) INIA 302 Amarilis potato.

A similar pattern was observed in Sulluscocha–Namora, where significant differences among treatments were also detected (Figure 2). TR-2 again showed the lowest severity (26.6% ± 1.6%), followed by TR-4 (agricultural lime) with 26.6% ± 6.0%. Conversely, the control treatment (TR-1) reached the highest severity (80.0% ± 5.0%), representing nearly three times the damage observed in TR-2 (Figure 2 and Figure 3, Table S1).

### 3.3. Number of Live Larvae in Sulluscocha and Cochapampa

**Figure 4:**
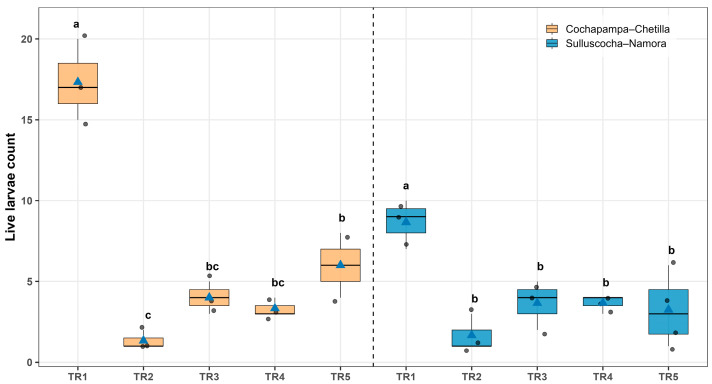
Number of potato tuber moth (*Symmetrischema tangolias*) larvae in stored potato tubers under five treatments: TR1 (control), TR2 (*Bacillus thuringiensis* var. *kurstaki* plus industrial talc), TR3 (industrial talc), TR4 (agricultural lime), and TR5 (wood ash), evaluated in two localities: Cochapampa–Chetilla (orange) and Sulluscocha–Namora (blue). Boxes represent the interquartile range, the horizontal line within each box indicates the median, blue triangles correspond to the mean, and grey dots represent individual observations. Different lowercase letters above the boxes indicate significant differences among treatments according to the Šidák test (*p* < 0.05).

Significant differences in the number of *Symmetrischema tangolias* larvae were observed among treatments in both localities (Figure 3). In Cochapampa–Chetilla, TR-2 (*Bacillus thuringiensis* var. *kurstaki* + industrial talc, 5 kg t^−1^ of seed) resulted in the lowest larval count (1.3 ± 0.3), followed by TR-4 (agricultural lime) with 3.3 ± 0.3. In contrast, the control treatment (TR-1) recorded the highest larval abundance (17.30 ± 1.45), nearly three times greater than that observed in TR-2 (Figure 4, Table S1).

A similar trend was recorded in Sulluscocha–Namora, where TR-2 again yielded the lowest larval number (1.6 ± 0.6), followed by TR-3 (industrial talc) and TR-4 (agricultural lime) with 3.6 ± 0.8 and 3.6 ± 0.3, respectively. Conversely, the control treatment (TR-1) exhibited the highest larval density (8.6 ± 0.8), approximately three times higher compared with TR-2 (Figure 4, Table S1).

## 4. Discussion

Our findings demonstrate that a powdered formulation of *Bacillus thuringiensis* (Bt) mixed with inert talc can dramatically suppress potato tuber moth infestations during storage. The Bt-talc treatment led to the lowest incidence of tuber damage and larval abundance among all interventions, significantly outperforming both the untreated control and the inert dusts alone. This result aligns with numerous studies showing that Bt-based dusts are highly effective against potato tuber moths in postharvest settings. For instance, Kroschel and Koch [24] achieved about 96% control of *P. operculella* by dusting stored tubers with a mixture of Bt (subsp. *kurstaki*) and fine sand. Likewise, field trials in the Indian Himalayas reported that dusting seed potatoes with a high-potency Bt powder (10^9^ spores/g) provided *complete* protection against tuber moth for at least 2 months [32]. After 90 days of storage, tubers treated with either Bt or the granulosis virus (another biocontrol) had only ~5–9% infestation, compared to 78–94% in untreated controls [32]. These parallel successes underscore that our Bt-talc approach is not an outlier; rather, it corroborates a broad consensus that Bt-based biopesticides can rival or exceed chemical insecticides in protecting stored potatoes [33,34,35,36]. Notably, a study in Nepal found Bt as effective as deltamethrin (a synthetic pyrethroid) in reducing tuber moth damage in storage [33]. The mode of action of Bt in storage is critical: dusted *B. thuringiensis* spores and toxins coat the tuber surface and are ingested by neonate larvae as they attempt to penetrate the potato, leading to gut infection and larval death before significant mining damage occurs [34,35]. This targeted activity likely explains the superior performance of the Bt-talc treatment in our trial. It is worth noting, however, that effective coverage of tubers is essential. Some reports suggest that if tubers are not thoroughly coated, or under heavy initial infestations, Bt alone may not prevent all damage (e.g., one study observed residual tuber damage despite Bt dust treatment) [33,35]. In our case, the high efficacy of Bt-talc indicates that the formulation and application method were sufficient to ensure lethal exposure to the vast majority of larvae.

The strong protective effect of agricultural lime dust observed in our study further highlights the potential of inert mineral powders in postharvest moth control. Agricultural lime (finely ground calcium carbonate) was the second-best treatment, significantly reducing infestation levels relative to the control. This finding is consistent with prior research showing that certain mineral dusts can directly cause high mortality of tuber moth larvae. For example, Schaub and Kroschel [36] reported that pure calcium carbonate dust caused up to ~95–99% larval mortality of the Guatemalan potato tuber moth (*Tecia solanivora*) under experimental storage conditions. In their study, CaCO_3_ alone was so effective against *T. solanivora* that adding biocontrol agents yielded little further benefit. The mode of action of lime and similar dusts is principally physical and physiological: fine particles abrade the insect cuticle and absorb cuticular waxes, desiccating the larvae and impeding their movement [37]. Additionally, lime’s alkaline nature may create a mildly caustic environment deterring eggs and larvae. Traditional Andean farmers have long exploited such effects; mid-20th-century trials in India showed that covering stored potatoes with layers of wood ash and lime could prevent potato tuber moth damage [32]. Similarly, in Peru, farmers and researchers experimented with dusts like ash, lime, and charcoal as non-chemical protectants [38,39]. Some earlier tests yielded only partial success, for instance, one report noted that neither ash nor lime alone completely protected tubers from *Symmetrischema tangolias* (the Andean potato tuber moth) under severe pressure [33]. Such variability in outcomes likely depends on application quality, pest pressure, and environmental conditions. High humidity, in particular, can diminish the efficacy of mineral dusts by causing clumping and reduced desiccation. Schaub and Kroschel [36] observed that the performance of CaCO_3_ dust dropped from ~98% kill to ~55% when relative humidity reached 100%. Despite these caveats, the weight of evidence (including our results) indicates that dry powdery minerals like lime can substantially curb tuber moth infestation in storage, especially in arid or well-ventilated stores where the dust remains effective.

In addition to these physical mechanisms, our findings align with broader evidence indicating that alkaline mineral dusts may also interfere with egg development. Laboratory studies on a range of insects show that extreme pH conditions can significantly reduce egg viability and delay or prevent hatching. For instance, Hyelemad et al. [40] demonstrated that both acidic and alkaline pH levels alter embryogenesis duration and substantially reduce hatchability in *Culex quinquefasciatus*. Similarly, Johnson et al. [41] reported that combinations of suboptimal pH, low moisture, and drought sharply decreased egg survival of the clover root weevil in soil environments. Complementary evidence from Pangihutan et al. [42] shows that mineral powders such as talc, calcium oxide and calcium hydroxide significantly suppress fruit fly oviposition and egg deposition on chili fruits. Together, these studies support the plausibility that the strongly alkaline microenvironment generated by agricultural lime and lime-rich ashes on treated tubers may hinder egg hatching or early larval establishment in potato tuber moths. In parallel, our Bt-talc treatment is consistent with evidence that talc-based formulations enhance the adhesion and persistence of Bt spores. Bouslama et al. [43] found that a CMC talc Bt formulation maintained high efficacy and persistence even after simulated rainfall, while Bravo et al. [44] emphasized that the ingestion of viable Cry-containing crystals is essential for larval mortality. Thus, the superior efficacy of our Bt-talc treatment reflects the convergence of two mechanisms: the intrinsic physiological vulnerability of neonate larvae to ingested Bt toxins and the improved deposition, adhesion, and persistence of Bt propagules enabled by the talc carrier.

Wood ash, another treatment evaluated in our study, showed a moderate level of protection, inferior to Bt-talc or lime, but still better than no treatment. This outcome is in line with the mixed but generally positive record of ash in the literature. Wood ash is a readily available by-product in farming communities and has historically been used as a natural protectant for stored produce. Fine wood ash dust can act similarly to other inert dusts by desiccating and mechanically interfering with insects [45,46,47]. Indeed, extension publications and reviews note that dusting potato tubers with fine ash can “control infestations for long periods” in storage. In parts of Africa and Asia, farmers store seed potatoes in layers of ash or periodically rub ash on tubers to deter tuber moths and other pests [48,49]. Our finding that ash alone did not completely prevent infestation is not unexpected; while ash can reduce pest survival, its efficacy tends to be lower than more chemically active dusts. For example, von Arx & Gebhardt [50] found that neither ash nor sand alone provided full protection in Peru’s central highlands, necessitating supplemental measures. Ash particles are lighter and less abrasive than silica-rich dusts, and they may drop off tubers over time, which could lead to some larvae surviving or eggs being laid on exposed spots. Nonetheless, ash’s partial effectiveness can still be valuable in an integrated approach, especially for resource-limited farmers. It is cheap, safe, and can be combined or alternated with other tactics. Some studies have suggested that ash works best when used in thick layers or in combination with other substances. For instance, Lal [51] reported enhanced control when wood ash was mixed with lime and used as a covering layer on stored potatoes [33]. In our context, the ash treatment likely provided a drying layer on the tubers, which slowed down infestation buildup, although it could not match the more targeted toxicity of Bt or the stronger desiccation of lime. Future work could explore whether enriching wood ash with small amounts of lime or biopesticides (as a combined formulation) might improve its protective efficacy without sacrificing its local availability and low cost.

The inclusion of industrial talc alone (without Bt) in our trial allowed us to gauge the inherent effect of an inert carrier dust on tuber moths. As expected, talc by itself was the least effective dust treatment, offering only limited pest suppression. Talc is a hydrophobic magnesium silicate with very fine, smooth particles; unlike abrasive dusts (ash, sand, diatomaceous earth) or alkaline dusts (lime), pure talc has minimal direct toxicity to insects. Any protective effect from talc is likely due to a mild physical barrier on the tuber surface, which might obstruct some neonate larvae or discourage oviposition to a small extent. In the literature, other inert powders of a similar nature have shown modest results unless combined with biocides. For example, research in Yemen and Ecuador found that talcum powder or kaolin clay alone could cause significant mortality in *P. operculella* larvae (ranging roughly 76–90% kill in laboratory tests), but these inert powders performed markedly better when formulated as carriers for agents like Bt or granulovirus [36]. Kroschel et al. [24] specifically chose fine sand/talc as a Bt carrier to improve adhesion to tubers and contact with larvae, achieving near-complete control only with the Bt toxin present [52]. Consistent with those insights, our talc-only treatment showed that, in the absence of Bt, an inert dust provides incomplete protection at best. This reinforces the view that while mineral dusts create an unfavorable environment for tuber moths, they are most effective when acting synergistically with a biocidal agent. It is also noteworthy that storage conditions can influence an inert dust’s standalone efficacy. In extremely dry conditions, even a relatively inert powder might desiccate more larvae, whereas in more humid storerooms, its effect would be negligible. Thus, the primary value of the talc in our study was as a benign carrier to deliver Bt uniformly on the tubers, a role it fulfilled well in the Bt-talc combination, judging by that treatment’s pronounced success.

These observations are consistent with the physicochemical behavior of fine mineral powders such as talc. Molecular simulations demonstrate that although talc can adsorb isolated water molecules at low relative humidity, at high humidity or near saturation, the cohesive interactions among water molecules dominate, preventing full wetting of the mineral surface [53]. As a result, water tends to bead on talc-like surfaces rather than forming continuous liquid films, which strongly limits humidity-induced caking or agglomeration. This behavior is consistent with our field observations, where the thin dust layer with a particle size range of 0.1–20 µm applied to seed tubers showed no signs of moisture-driven aggregation under protected storage conditions.

There is evidence that *S. tangolias* (often called the Andean potato tuber moth) can be somewhat less susceptible to inert dust treatments than *P. operculella*. Schaub and Kroschel [36] observed that while a plain calcium carbonate dust was very potent against *T. solanivora* (another potato tuber pest) and *P. operculella*, it was less so against *S. tangolias*, leading those authors to recommend adding 15 g of Btk per kg dust specifically to bolster *S. tangolias* control. In our trial, the clear superiority of the Bt-infused talc over plain talc or ash underscores the value of combining modes of action, particularly when multiple tuber moth species are present. Likely, *S. tangolias*’ behaviour or biology (perhaps faster penetration into tubers, or more robust larvae) makes it harder to kill with contact desiccants alone, hence the observed benefit of Bt’s gut-active toxin for that species. The performance of agricultural lime in our study against both moth species is encouraging, but it may also reflect the relatively cool, dry conditions of highland potato stores, which favor dust efficacy. In warmer, more humid lowland storage, purely inert measures might struggle against *S. tangolias*, reinforcing the idea of integrating a biopesticide for reliable control. Overall, our data and the literature concur that an integrated dust + biocontrol approach provides a robust defense that covers the spectrum of potato tuber moth pests.

Taking a broader perspective, the convergence of our findings with those from diverse regions (South America, South Asia, Middle East) points to the considerable promise of integrating *B. thuringiensis* and mineral dusts into sustainable postharvest pest management. Both components of this strategy have strong environmental and safety profiles. Bt is a naturally occurring bacterium used globally in biopesticides with minimal non-target impacts, and inert dusts like lime or ash are non-toxic to humans and leave no harmful residues on seed tubers. This makes them especially suitable for seed potatoes, which must be stored without chemical contamination that could affect germination or farmer safety. Moreover, these treatments are compatible with other sustainable practices. In smallholder systems in the Andean highlands, seed tubers are typically stored in simple, non-climate-controlled structures with diffuse light and natural ventilation. Tubers are placed over wooden pallets, straw, or other insulating materials to avoid direct contact with the ground, and arranged in no more than three layers. Depending on the pallet size, 100–120 kg of seed can be stored per square meter. These traditional conditions facilitate moth infestations and highlight the importance of storage hygiene. For example, tuber moth control can be enhanced by (a) improved storage hygiene (removing infested tubers, using sealed or netted containers to exclude moths), and (b) complementary biocontrol agents such as parasitoids or viruses. While chemical insecticides can suppress tuber moths, they carry risks of resistance, user exposure, and regulatory restrictions on seed use; in contrast, a Bt + dust approach can be applied repeatedly without these drawbacks. In practice, a combined strategy might involve dusting seed tubers with a formulation of Bt in lime (or talc) at storage onset, and possibly reapplying a small amount of dust periodically or when new tubers are added, to maintain a protective barrier. Farmers in the Andes could even produce their own dust blends (e.g., mixing commercially available Bt powder with sifted wood ash or lime) as a low-cost alternative to synthetic pesticides.

Despite the demonstrated efficacy of Bt-talc and mineral dusts, adoption among Andean smallholders remains low. This is largely because Bt-talc is a relatively new alternative for farmers, and its widespread use will depend on effective technology transfer and demonstration at the community level. The estimated cost of treating one metric ton of seed potatoes is approximately USD 40, which includes both the Bt-talc product and the labor required for its application (two worker-days). While this cost is modest compared with repeated chemical sprays, farmers may still be hesitant to adopt a technology that is unfamiliar or not yet widely validated in on-farm settings. Therefore, scaling efforts and farmer training will be essential to accelerate adoption and ensure that the benefits observed in experimental settings translate into real-world practice. Notably, our study and others have shown that such treatments can drastically cut losses over long storage periods (3–5 months) [32], which is the critical window for seed preservation until the next planting. In conclusion, the integration of a bioinsecticide like *B. thuringiensis* with inert mineral dusts represents a practical and effective strategy for postharvest pest management in potatoes. This approach leverages synergistic effects, the immediate physical protection from dust and the lethal biological action of Bt, to achieve high levels of control. Our results contribute to a growing body of evidence that these tools, used in combination, can protect stored potatoes in a manner that is economically feasible for farmers and aligned with the principles of sustainable agriculture. Embracing such biocontrol-based methods will help reduce reliance on chemical fumigants or insecticidal sprays in potato stores, thereby promoting safer storage practices. Future on-farm trials and scaling efforts are warranted to refine the application techniques (e.g., optimal dust layering, formulation ratios) and to educate farmers on the benefits. If widely adopted, Bt and mineral dust treatments could become a cornerstone of integrated pest management (IPM) for stored potatoes, preserving yield and seed quality while safeguarding farmer health and the environment [36]. The success of this approach in highland Peru may serve as a model for other potato-growing regions seeking to implement more sustainable postharvest insect control solutions.

Although our findings demonstrate the strong potential of Bt-talc and agricultural lime for postharvest control of potato tuber moths, several limitations should be acknowledged. First, the experiment was conducted exclusively on the variety INIA 302 Amarilis; given that skin texture, periderm thickness, and chemical composition vary among potato cultivars, the applicability of our dust-based scheme to other varieties remains to be validated. Second, we did not assess treatment performance under different initial pest population densities. Future studies should incorporate gradient infestation levels to identify control thresholds and determine how dust efficacy scales with pest pressure. Third, while dust treatments are widely regarded as safe, we did not quantify potential agronomic side effects, such as impacts on tuber germination or whether residual agricultural lime alters soil pH at planting. A more comprehensive safety assessment, including germination tests and soil pH monitoring, would strengthen the practical recommendations derived from this work. These limitations highlight the need for broader, multi-varietal and multi-scenario evaluations before large-scale adoption of the proposed control scheme.

## 5. Conclusions

This study demonstrated that the application of *Bt-talc* (*Bacillus thuringiensis*) at a dose of 5 kg t^−1^ of seed is an ecologically effective strategy for controlling *Symmetrischema tangolias* and *Phthorimaea operculella* under storage conditions, as it significantly reduced incidence, severity, and the number of live larvae. Agricultural lime also showed comparable effectiveness and, due to its lower cost and higher availability, represents a viable alternative for smallholder farmers in high-Andean regions with limited access to commercial inputs. Looking forward, the integration of these approaches into pest management programs could strengthen the sustainability of potato production systems, reduce dependency on synthetic pesticides, and promote safer and more resilient agricultural practices in rural Andean communities.

## Figures and Tables

**Table 1:** Description of the treatments under study.

Treatment	Description	Specific Properties
TR-1	Control	
TR-2	*Bacillus thurigiensis* var. *Kurstaki* (15 g) plus industrial talc (1000 g)	Soft texture; particle size Ø 0.1–10 µm
TR-3	industrial talc	Soft texture; particle size Ø 0.1–10 µm
TR-4	Agricultural lime	Purity > 85% particle size Ø 0.1–20 µm
TR-5	Wood ash	Sourced from *Eucalyptus globulus*

## Data Availability

The data are available by contacting the corresponding authors for collaboration.

## Supplementary Materials

The following supporting information can be downloaded at: https://www.mdpi.com/article/10.3390/insects17010086/s1, Figure S1: Geographic location of the experimental sites managed by the Baños del Inca Agrarian Experimental Station. (A) Sulluscocha–Namora and (B) Cochapampa–Chetilla; Figure S2: Monthly rainfall, maximum and minimum temperature, and relative humidity recorded during the experimental period (2024–2025) in (A) Sulluscocha–Namora and (B) Cochapampa–Chetilla; Table S1: Incidence (%), severity (%), and live larvae count for five treatments in Cochapampa and Sulluscocha during 2024–2025.
