# Supplementary material for: Sustainable Management of Potato Tuber Moths Using Eco-Friendly Dust Formulations During Storage in the Andean Highlands
Source: Insects. 2026 Jan 13;17(1):86. doi: 10.3390/insects17010086 (PMC12842296; doi:10.3390/insects17010086)
Supplement: Supplementary file 1 [file insects-17-00086-s001.zip › insects-4004670-supplementary.pdf]

Supplementary Figure and table

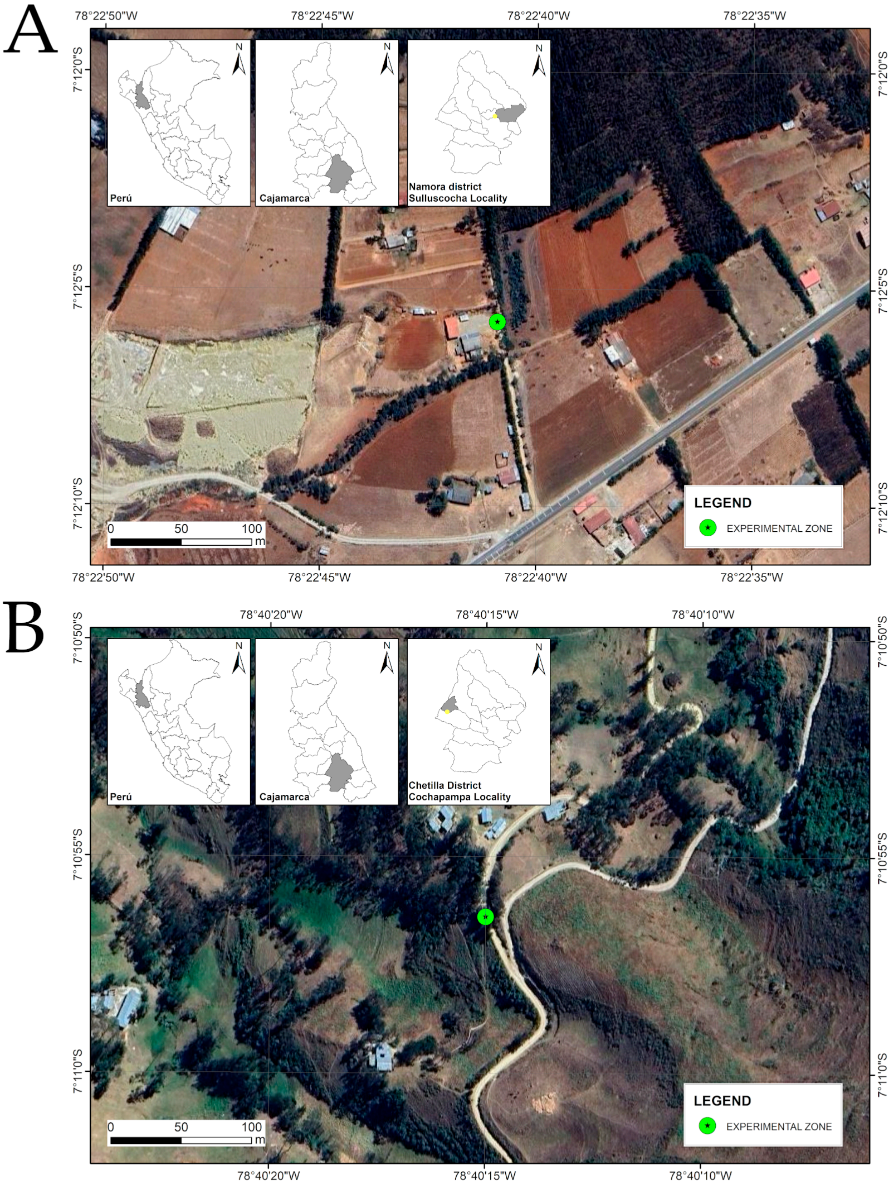

Figure S1. Geographic location of the experimental sites managed by the Baños del Inca Agrarian Experimental Station. (A) Sulluscocha–Namora and (B) Cochapampa–Chetilla.

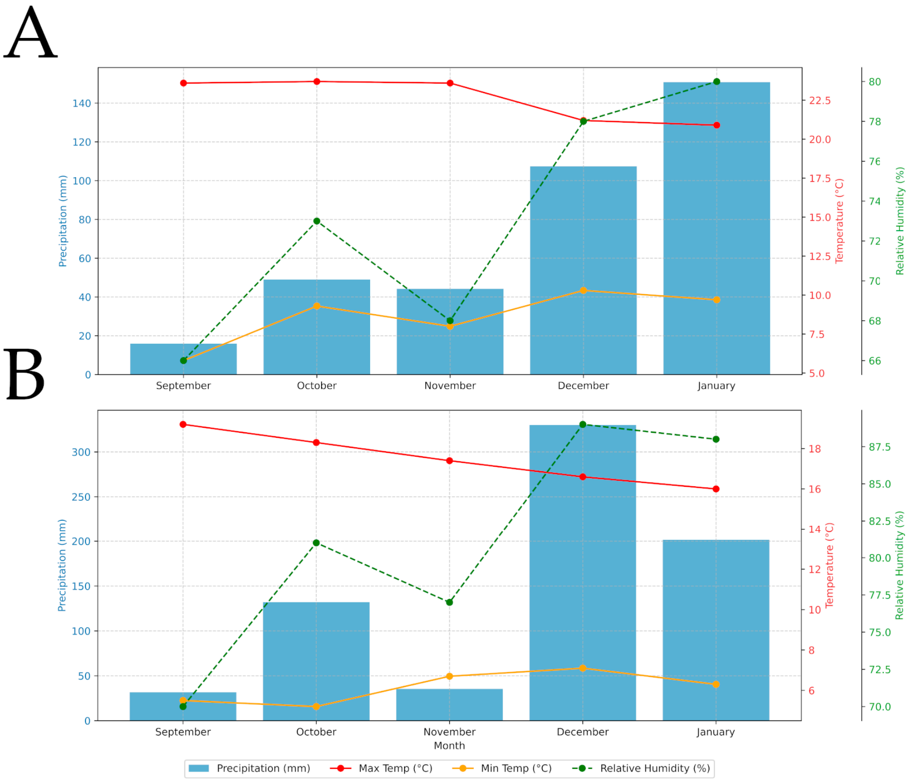

Figure S2. Monthly rainfall, maximum and minimum temperature, and relative humidity recorded during the experimental period (2024–2025) in (A) Sulluscocha–Namora and (B) Cochapampa–Chetilla.

Table S1. Incidence (%), severity (%), and live larvae count for five treatments in Cochapampa and Sulluscocha during 2024–2025.

Treatment	Location	Incidence (%)	Severity (%)	Live Larvae count
		Mean ± S.E	Mean ± S.E	Mean ± S.E
TR1	Cochapampa	58.10% [a] ± 3.95%	63.3% [a] ± 9.28%	17.3 [a] ± 1.45
TR1	Sulluscocha	64.23% [a] ± 3.04%	80.0 %[a] ± 5.00%	8.67 [a] ± 0.88
TR2	Cochapampa	16.80% [c] ± 6.26%	16.67% [b] ± 1.67%	1.33 [c] ± 0.33
TR2	Sulluscocha	25.55% [c] ± 4.86%	26.61% [c] ± 1.69%	1.67 [b] ± 0.67
TR3	Cochapampa	26.10% [bc] ± 6.27%	21.67% [b] ± 1.67%	4 [bc] ± 0.57
TR3	Sulluscocha	37.23% [bc] ± 4.06%	33.33% [bc] ± 4.40%	3.67 [b] ± 0.88
TR4	Cochapampa	23.95% [c] ± 8.44%	21.67% [b] ± 4.41%	3.33 [bc] ± 0.33
TR4	Sulluscocha	30.42% [c] ± 3.19%	26.67% [c] ± 6.00%	3.67 [b] ± 0.33
TR5	Cochapampa	50.61% [ab] ± 1.30%	53.33% [a] ± 8.82%	6 [b] ± 1.15
TR5	Sulluscocha	51.1% [ab] ± 3.36%	51.66% [b] ± 7.26%	2.33 [b] ± 0.88

* SE: Standard Error.
